# Dentifrices

**Published:** 1887-04

**Authors:** 


### DENTIFRICES.

*The Pharmaceutical Record,* not long since, contained an excellent article upon "Tooth Preparations," as viewed from the druggists' standpoint. There is no disputing the fact that very crude ideas exist among dentists, concerning the ingredients that should enter into the composition of a proper tooth powder. And yet the importance of the proper preparation of a dentifrice cannot easily be overestimated. When we reflect that under the stimulus of the warmth, moisture and presence of many fermentive organisms in the human mouth, food will undergo chemical changes in a very short time, the importance of keeping the oral cavity clean becomes apparent. There is many a housewife who will scrupulously wash and dry each utensil employed in cooking every time it is used, will thoroughly cleanse every plate upon the table after each meal, but who thinks that she has fulfilled her duty faithfully when she has once a day cleaned the teeth with which she eats. She would not let her children use a spoon that had not been washed, yet they are allowed to use their teeth for an indefinite time without scrubbing. At the most, from one meal to another the remains of food would only dry down upon the hard plate, while it will rot in the mouth during the same time. How thoroughly do such people illustrate the scripture adage concerning those who "make clean the outside of the cup and the platter," but whose inward part is full of all impurity.

The teeth may be carefully washed with water, but this is not sufficient. Unless they be kept polished, it is impossible for them to be pure. A rusty knife is never clean, for the roughened surface affords lodgment for matter that cannot be washed away. The same rule holds good with the teeth. A dentifrice or powder is occasionally needed to keep the teeth polished. The name of those offered for sale is legion, and the more ignorant of the character of drugs a dentist is, the more is he possessed with a desire to invent a new tooth powder which shall contain all manner of incompatible ingredients.

A western dental journal, not long since, published the report of a discussion upon "The Hygienic Treatment of the Mouth and

Teeth" in a dental society which, however, met in an eastern and not a western city. Here is what one speaker said:

From my experience, my results as to tooth powders are these: I do not put in any honey or sugar. Honey is the worst thing you can put in, next orris-root and next sugar. These should not be used because they are fermentable, and so make sore mouths from constant irritation. They have no place in tooth powders. If you want to use something in place of sugar, use bicarbonate of soda, and biborate of soda instead; the latter is a benefit and does no harm. Use glycerine if you wish a paste; it does not ferment in the mouth. In place of orris-root, grind up cloves. Use cassia or sassafras bark; these do not ferment, as their essential oils prevent. Yellow or red Peruvian bark is good. In making a powder I would use floated chalk instead of precipitated chalk.

Question. How do you get floated chalk?

Answer. I throw the chalk on the water and gather that which floats, and throw away all that readily precipitates. My formula for a good tooth powder is this: Take of floated chalk $\frac{1}{3}$, bicarbonate of soda and biborate of soda, each $\frac{1}{8}$; salicylic acid, $\frac{1}{50}$; extract cinchona, $\frac{1}{33}$; oil cloves, $\frac{1}{50}$; oil sassafras, $\frac{1}{50}$; and oil cassia, $\frac{1}{50}$. Subject passed.

Well, it was time it was passed. Here is one who pretends to stand as a teacher of others, and who asserts that certain articles should not be used in a dentifrice "because they are fermentable and so make sore mouths." And what, pray, are the "fermentable" substances to which this wiseacre objects? First, he "does not put in any honey or sugar." The sugar that is used in tooth powders is always cane sugar, and any one who knows anything of physiology knows that this is entirely unfermentable until it is changed to grape sugar, and this process requires some time to effect. Honey is readily soluble in the fluids of the mouth, as such ingredients of a good powder should be, and hence it could not ferment if properly used. After honey, this wonderful chemist considers orris-root as most objectionable, because of its liability to fermentation. There did not seem to be one present at the meeting who could correct such an absurd assertion. Orris-root cannot ferment, for it is not a fermentable substance. Bicarbonate or biborate of soda are not ingredients in any good tooth powder, because they have no virtues that specially recommend them for such a purpose.

"Floated chalk," or the process of obtaining it, as given by the speaker, is very funny. If he wants chalk he should use "precipitated," which is a chemical product, and is free from every objectionable material. He would "float" his chalk to rid it of heavy or silicious matter, while precipitated chalk is chemically free from it.

The "formula" given is a curiosity. The apothecary who should endeavor to make up a powder from it would require to be a mathematician indeed. All of the proportions added together do not make an integer, and to compound this formula the chemist must each time take a fraction of an indefinite fraction, and when he is through he will have but a fraction as the result. Why will dentists assert such nonsense in the face of the world, and thus bring ridicule upon the societies of which they form a part? Why will men assume a knowledge which they do not possess, when their ignorance cannot fail of an exposure?

The amount of the whole is, dentists have no business to attempt the compounding of a tooth powder for common use. In special cases of diseased mouth, those who are quite competent should write a prescription to be prepared by a druggist, but the making of a proper dentifrice requires special facilities, which are not at the command of the average apothecary. Many years ago, Dr. I. W. Lyon, of New York, a regular graduate in dentistry, concluded to devote his exclusive attention to the manufacture of a dentifrice which should be approved by the best dentists. The formula was made public, and Dr. Lyon has continued to manufacture from it to this day, using only the best drugs and himself attending to their preparation, which is done by aid of perfect machinery. Nor is his the only good dentifrice manufactured. The S. S. White Dental Manufacturing Company make a number, which dentists can purchase. Lyon's powders are kept by all respectable druggists. How much more professional it is for a dentist, when he would order an ordinary powder, to write a prescription for one that is standard, than to attempt the compounding of such absurd formulas as that given above.

